# Social Anxiety, Depression and Self-Esteem in Obese Adolescent Girls with Acanthosis Nigricans

**DOI:** 10.4274/jcrpe.1515

**Published:** 2015-03-05

**Authors:** Özgür Pirgon, Gonca Sandal, Cem Gökçen, Hüseyin Bilgin, Bumin Dündar

**Affiliations:** 1 Süleyman Demirel University Faculty of Medicine, Department of Pediatric Endocrinology and Diabetes, Isparta, Turkey; 2 Süleyman Demirel University Faculty of Medicine, Department of Neonatology, Isparta, Turkey; 3 Gaziantep University Faculty of Medicine, Department of Child and Adolescent Psychiatry, Gaziantep, Turkey; 4 Konya Training and Research Hospital, Clinic of Pediatrics, Konya, Turkey; 5 Katip Çelebi University Faculty of Medicine, Department of Pediatric Endocrinology and Diabetes, Izmir, Turkey

**Keywords:** Self-esteem scores, acanthosis, obesity, adolescent, hyperandrogenism

## Abstract

**Objective:**

To assess the impact of acanthosis nigricans (AN) on depression symptoms, related quality of life and self-esteem scores in obese adolescent girls.

**Methods:**

Fifty-nine obese adolescent girls (mean age: 13.19±1.3 years, age range: 12-17 years, mean body mass index: 29.89±3.30) were enrolled in this study. The obese adolescent girls were divided into two groups based on presence or absence of AN. Non-obese healthy adolescents constituted the control group (30 girls, mean age: 13.5±1.4 years). All subjects were evaluated using the Children’s Depression Inventory (CDI), the State-Trait Anxiety Inventory for Children (STAI-C), and the modified Rosenberg Self-Esteem Scale (SES). Higher scores indicated more severe depression and anxiety, as well as low self-esteem status.

**Results:**

The AN and non-AN obese groups showed significantly higher CDI, STAI-C and SES scores than the control group, and the two obese groups demonstrated no significant differences for these scores. The AN obese group with higher total testosterone levels (>50 ng/dL) had higher scores for SES (2.55±1.8 vs. 1.42±1.2; p=0.03) than the AN obese group with low total testosterone levels. SES scores significantly correlated with total testosterone levels (r=0.362; p=0.03) and fasting insulin (r=0.462; p=0.03) in the AN obese group.

**Conclusion:**

Higher SES scores (low self-esteem status) were determined in obese adolescents with acanthosis and were related to hyperandrogenism. This study also showed that a high testosterone level may be one of the important indicators of low self-esteem status in obese girls with AN.

## INTRODUCTION

The consequences of childhood overweight include increased risk of adult obesity in addition to greater morbidity and mortality as adults (1). Increased oxidative stress, insulin resistance, acanthosis nigricans (AN) and functional ovarian hyperandrogenism are all conditions associated with obesity (2,3).

AN is a skin condition marked by darkening and thickening of the skin into irregular folds (4). People who are obese can develop AN, and it is also related to endocrine disorders such as hyperandrogenism and obesity-related insulin resistance (5). The markings on the affected skin are very visible and may have an unpleasant smell (6). Adolescence is an age period in which physical changes and appearance occupy an important place in the self image of an individual. For this reason, obesity with acanthosis may predispose to negative psychosocial consequences in an adolescent.

Adolescence is a critical developmental phase characterized by dramatic physical and psychological changes (7). The last decade witnessed an increasing number of studies examining the relationship between obesity and depression among adolescents (8,9,10,11). Although low self-esteem has been found to be related to serious outcomes such as depression, suicidality, eating disorders and substance abuse (12,13), there is no report on the relationship between obesity-related dermatologic conditions (such as AN) and their psychosocial consequences in adolescents. The purpose of this study was to examine the effect of AN on depression, social anxiety and self-esteem in obese female adolescents.

## METHODS

**Figure 1:**
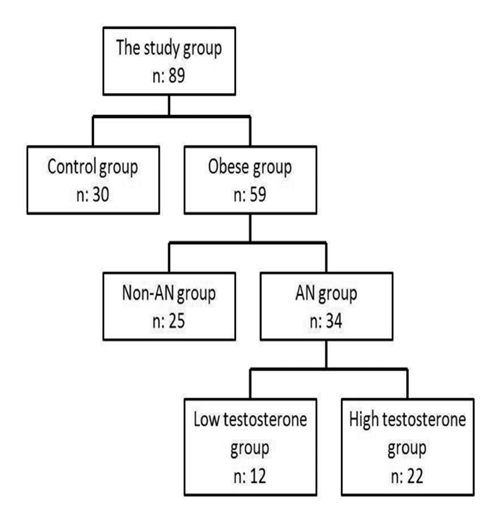
The classification of the subjects

Fifty-nine female obese adolescents with a mean age of 13.19±1.3 years (range 12-17 years) and a mean body mass index (BMI) of 29.89±3.30 were recruited from among obese children who were admitted to our Pediatric Endocrinology Unit between April 2011 and April 2012. The obese group was divided into two subgroups: (1) AN group (34 girls, mean age: 14.4±1.5 years, mean BMI: 28.7±4.7); the diagnosis of AN confirmed by an experienced pediatric dermatologist and (2) non-AN group (25 girls, mean age: 13.9±1.4 years, mean BMI: 28.4±3.6) without any evidence of dermatologic changes in the axillary region. The AN group was further divided into two subgroups according to their total testosterone levels (a total testosterone level >50 ng/dL was accepted to indicate hyperandrogenism) (Figure 1). The control group included 30 pubertal stage-matched non-obese healthy girls with a mean age of 13.5±1.4 years (age range 12-17) and a mean BMI of 20.2±3.2. Patients were excluded if they had been diagnosed with a disease, including polycystic ovary syndrome (signs such as acne, hirsutism and menstrual irregularity), type 1 or type 2 diabetes mellitus, took medications or had a condition related to insulin action or insulin secretion (e.g. glucocorticoid therapy, hypothyroidism, Cushing’s disease). The study protocols were approved by the institutional review board of Konya Research Hospital Ethics Committee. Signed informed consent was obtained from the parents of the children.

Anthropometric measurements were performed in all patients. Height and weight were measured with an empty bladder and in the postabsorptive state. Height was measured to the nearest 0.5 cm on a standard height board and weight was determined to the nearest 0.1 kg on a standard physician’s beam scale with the subject dressed only in light underwear without shoes. BMI was calculated as weight (in kilograms) divided by height (in meters) squared. Patients with a BMI of ≥95th percentile according to the reference curves for Turkish adolescents were accepted as obese. Pubertal development stage was assessed by a single pediatric endocrinologist using the Tanner criteria. Staging for sexual maturation was >2 in all patients (Tanner stages 2-5).

Fasting blood samples (at 8:00 am) were obtained to measure serum glucose, insulin, and total testosterone levels in the morning by venipuncture after an overnight fast (at least 12 h). Glucose was determined by the glucose oxidase method. Serum concentrations of total cholesterol, high-density lipoprotein cholesterol (HDL-cholesterol) and triglycerides were measured using routine enzymatic methods with Abbott diagnostics C16000 chemistry analyzer. Serum insulin levels were measured by an Immulite immunoassay (Diagnostic Products, Los Angeles, CA, US). Insulin resistance was estimated using the homeostasis model assessment for insulin resistance (HOMA-IR); fasting insulin concentration (µU/mL) x fasting glucose concentration (mg/dL)/405 (14). Insulin resistance was defined in adolescents as HOMA-IR levels greater than 3.16 (15). In this study, we defined biochemical hyperandrogenism as a serum total testosterone above the normal reference range for Tanner stage 5 on a commercially available high-performance liquid chromatography/mass spectrometry (HPLC/MS) assay, reported as a total testosterone >50 ng/dL (16).

### Psychological Measurements

The Children Depression Inventory (CDI) is a self-report depression scale for children aged 9 years and over, modeled by Kovacs (17) after the Adult Beck Depression Inventory. Twenty-seven multiple-choice items assess the severity of depressive symptoms. The scale is widely used and it has demonstrated good concurrent validity and reliability in Turkey (18). Higher scores (min: 0; max: 54) represent more severe depression, and scores below 19 are considered to be normal.

The State-Trait Anxiety Inventory for Children (STAI-C), developed by Spielberger, has two subscales (19). The first one, state anxiety, defines the experienced anxiety under certain conditions and at a certain time; it changes according to external factors. The second is trait anxiety, which defines the feelings of the individual in general anwd reflects their general predisposition to anxiety. The validity and reliability study of the Turkish version was carried out by Ozusta (20).

Self-esteem was measured by using the Rosenberg Self-Esteem Scale (SES). The Rosenberg SES is a widely used measure of global self-esteem in adolescents (21). The reliability and validity of the instrument for Turkish adolescents has been established by Cuhadaroglu (22) This self-report instrument consists of 10 items rated on a 4-point Likert scale, with responses ranging from strongly agree to strongly disagree. We defined low self-esteem as above the median (greater than 2, with high scores indicating lower self-esteem) as described by Cuhadaroglu, albeit with reverse coding. Higher scores represent low self-esteem (0-1 points: high; 2-4 points: intermediate; 5-6 points: low self-esteem). Scores below 2 are considered to be normal.

### Statistical Analyses

Data are expressed as means ± SD. Mean and standard deviations were used as descriptive statistics. Differences in the means of variables were tested using nonparametric tests depending on the distribution of the variables. The correlations among numerical data were analyzed by the Pearson’s correlation coefficient (r). To analyze the associations between categorical variables, the Spearman’s rank correlation coefficient was used. A value of p <0.05 was used to indicate statistical significance.

## RESULTS

The characteristics of the study population are shown in Table 1. AN was most commonly found in the axillary region of the obese children. There was no significant difference among the AN and non-AN obese groups for BMI (p>0.05). All groups had normal serum total cholesterol, HDL-cholesterol and triglyceride levels and there were no significant differences in these parameters among the groups.

AN and non-AN obese adolescents had significantly higher fasting insulin levels (16.6±5.6 vs. 13.0±5.1 vs. 6.8±2.7 μU/mL), HOMA-IR values (3.85±1.45 vs. 2.98±1.22 vs. 1.53±0.63) and total testosterone levels (60.4±16.8 vs. 49.7±15.5 vs. 34.6±9.3 ng/dL) than the control group, as expected. However, there also was no significant difference between the obese groups (AN and non-AN) and the control group for fasting glucose levels (93.2±11.6 vs. 91.8±8.1 vs. 90.3±8.3 mg/dL) (Table 1). When the AN and non-AN subgroups were compared, the AN group was found to have higher fasting insulin levels (16.6±5.6 vs. 13.0±5.1 μU/mL), HOMA-IR values (3.85±1.45 vs. 2.98±1.22) and total testosterone levels (60.4±16.8 vs. 49.7±15.5 ng/dL) than the non-AN group.

We determined significantly higher scores of CDI, STAI-C1, STAI-C2 and SES in obese groups than the control group (Table 1). When we compared the AN and the non-AN obese group, we did not detect any difference in terms of CDI, STAI-C1, STAI-C2 and SES scores. However, when the AN group was divided into two subgroups according to their total testosterone levels, the AN group with high total testosterone had significantly higher SES scores and increased fasting insulin levels (19.6±6.5 vs. 14.9±4.4 μU/mL, p=0.01) than the AN group without hyperandrogenism (2.55±1.8 vs. 1.42±1.2; p=0.03) (Table 2).

**Figure 2:**
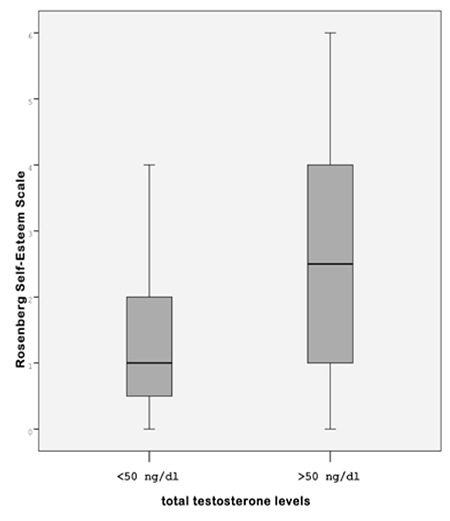
Comparison of the Rosenberg Self-Esteem Scale scores in obese girls with acanthosis according to total testosterone levels

Pearson’s correlation analysis was performed on the entire group of AN obese subjects with hyperandrogenism and control subjects to determine factors that correlated with SES scores. SES values were positively correlated with fasting insulin levels (r=0.462, p=0.03) and total testosterone concentrations (r=0.362, p=0.035) in the AN obese girls with hyperandrogenism and stepwise multiple linear regression confirmed that SES scores could be explained independently by total testosterone levels (Figure 2). SES scores were not found to be correlated with age, BMI, fasting glucose, HOMA-IR values in the AN obese group with hyperandrogenism (Table 3).

## DISCUSSION

We evaluated the depression, social anxiety and SES scores in AN and non-AN obese adolescents compared with control subjects. The findings demonstrated that SES scores were higher in AN and non-AN obese adolescents than the control group. Moreover, the girls with hyperandrogenism in the AN group had higher SES scores than the other groups. These data indicate that hyperandrogenism may lead to undesirable psychosocial consequences in obese adolescents with acanthosis.

Several evidence-based studies have shown that obese teens have a higher incidence of mental health problems such as depression, anxiety and poor self-esteem than their non-obese peers (23). Obesity is considered a pro-inflammatory state and increased inflammation and an altered stress system may be a common link between obesity and depression. A relationship between weight status and low self-esteem has also been demonstrated (8,9,10,11). However, results have been somewhat controversial, with some studies showing lower self-esteem among obese adolescents and others finding associations only among subgroups based on age or race, or for a particular domain of self-esteem. Franklin et al (8) found in a community sample of 2813 Australian youths that body dissatisfaction mediated the association between obesity and negative self-esteem in females. Swallen and colleagues (24) used a cross-sectional analysis of the 1996 National Longitudinal Study of Adolescent Health and found that among the youngest adolescents (aged 12 to 14 years), obesity was linked to depression and self-esteem. The result of this study suggested that the obese group had significantly higher CDI, STAI-C1, STAI-C2 and Rosenberg SES scores (high scores indicating lower self-esteem) than the control group. Low self-esteem has been associated with a number of psychological, physical, and social consequences that may influence successful adolescent development and the transition to adulthood.

Self-esteem, as an overall reflection of an individual’s self-worth, encompasses beliefs about oneself as well as an emotional response to those beliefs (25). Representing the capacity to feel worthy of happiness and to be able to successfully address life challenges, self-esteem is an important determinant of adolescent mental health and development (26). Self-esteem research has been conducted for several decades, but researchers have yet to reach a consensus on a definition for this construct. Several comprehensive assessment scales have been reported including the Rosenberg SES (21), the Piers-Harris Children’s Self-Concept Scale (9) and the Harter’s Self-Perception Profile. In this study, we used the Rosenberg SES modified by Cuhadaroglu for Turkish adolescents. In this scale, low self-esteem scores below 2 are considered to be normal (22). In our study, SES scores were higher in obese adolescents compared to the control group. In addition, higher testosterone levels were related to higher SES scores in the AN group.

We found a significant difference between the control group and both the AN and non-AN groups in terms of fasting insulin levels, IR and total testosterone concentrations. Several studies have reported positive associations between AN and hyperinsulinemia (27,28). AN is present in 90% of individuals with IR and in obese children (BMI >98th percentile), the prevalence is as high as 62%, regardless of race (29). The degree or severity of AN directly correlates with elevated circulating insulin levels in the blood (30). In the present study, SES scores were positively correlated with fasting insulin and total testosterone levels in AN obese girls with hyperandrogenism. Susman et al (31) found that higher levels of testosterone in the girls statistically accounted for the effect of pubertal status on depressive symptomatology. The recent publication on a possible relationship between excess androgens and psychological measures within the polycystic ovary syndrome population reported that lower body satisfaction (self-esteem and negative appearance scales) was associated with hyperandrogenism (32). Both the AN and non-AN groups showed no significant difference in the CDI, Rosenberg SES, STAI-C1 and STAI-C2. When the AN group was divided into two subgroups according to their total testosterone levels, the AN group with high total testosterone levels had higher scores for Rosenberg SES than the AN group with low total testosterone levels. This result indicates that hyperandrogenism has an important role in the negative psychosocial consequences observed in obese adolescents with AN.

In conclusion, physiological and psychological abnormalities are common in obese adolescents with AN. The most effective treatment for AN and low self-esteem is weight loss, through which the underlying condition of hyperinsulinemia can be corrected. Identification of modifiable risk factors for low self-esteem in adolescents is important in developing interventions to prevent the state of low self-esteem and to enhance self-esteem in this stage of life.

## Figures and Tables

**Table 1:**
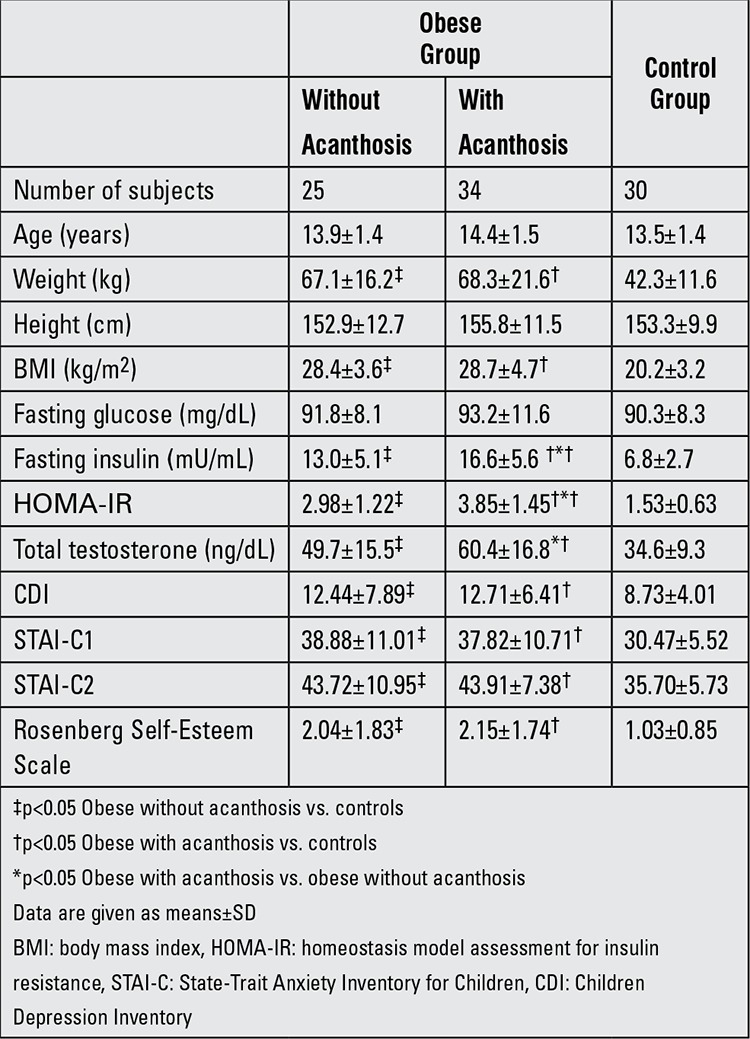
Characteristics of obese adolescent girls and control group

**Table 2:**
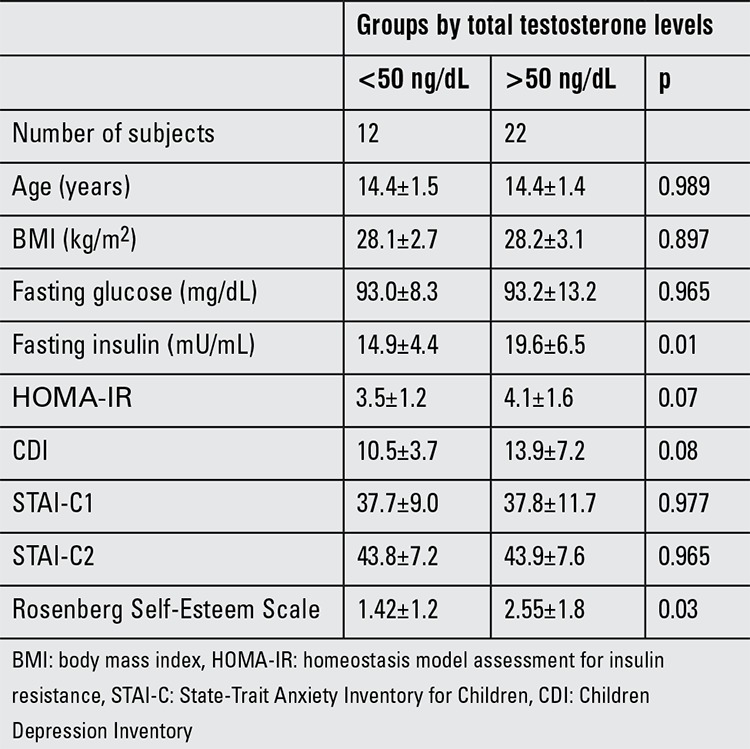
Comparison of social anxiety, depression and self-esteem scores according to total testosterone levels in the obese girls with acanthosis

**Table 3:**
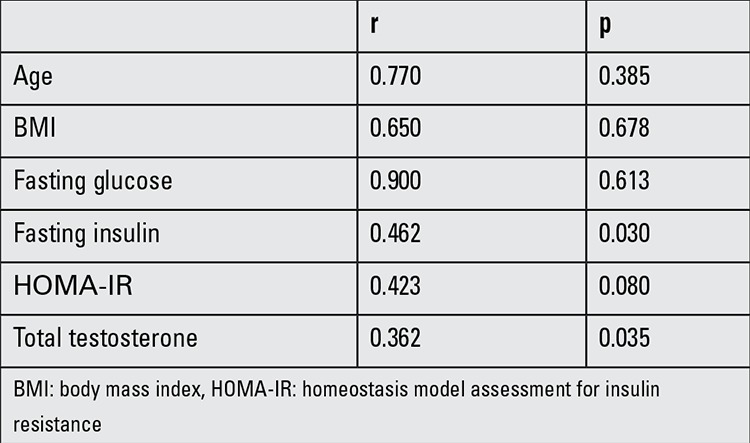
Pearson’s correlations between Rosenberg Self-Esteem Scale scores and the laboratory parameters in the acanthosis nigricans obese girls with hyperandrogenism (total testosterone levels >50 ng/dL)
